# Dietary Sources of High Sodium Intake in Turkey: SALTURK II

**DOI:** 10.3390/nu9090933

**Published:** 2017-08-24

**Authors:** Yunus Erdem, Tekin Akpolat, Ülver Derici, Şule Şengül, Şehsuvar Ertürk, Şükrü Ulusoy, Bülent Altun, Mustafa Arıcı

**Affiliations:** 1 Department of Internal Medicine, Division of Nephrology, Hacettepe University Faculty of Medicine, Ankara 06230, Turkey; yerdem@hacettepe.edu.tr (Y.E.); baltun@hacettepe.edu.tr (B.A.); 2 Department of Internal Medicine, Division of Nephrology, Istinye University Liv Hospital, Istanbul 34510, Turkey; tekinakpolat@yahoo.com; 3 Department of Internal Medicine, Division of Nephrology, Gazi University Faculty of Medicine, Ankara 06560, Turkey; ulver@gazi.edu.tr; 4 Department of Internal Medicine, Division of Nephrology, Ankara University Faculty of Medicine, Ankara 06100, Turkey; sule.sengul@medicine.ankara.edu.tr (Ş.Ş.); sehsuvarerturk@yahoo.com (Ş.E.); 5 Department of Internal Medicine, Karadeniz Teknik University Faculty of Medicine, Trabzon 61080, Turkey; sulusoy2002@yahoo.com

**Keywords:** blood pressure, epidemiology, hypertension, salt intake, urinary sodium, dietary sodium

## Abstract

Previous research has shown daily salt intakes in Turkey to be far above the recommended limits. Knowing the sources of dietary salt could form a basis for preventive strategies aimed towards salt reduction. This study aimed to investigate dietary sources of salt in Turkey. A sub-group (*n* = 657) was selected from the PatenT2 study population, which represented the urban and rural areas of 4 major cities (Ankara, Istanbul, Izmir, and Konya). A questionnaire inquiring about sociodemographic characteristics, medical histories, detailed histories of diet, and salt consumption was completed. Participants were asked to collect a 24-h urine sample and to record their food intake (dietary recall) on the same day. Of 925 participants selected, 657 (71%) provided accurate 24-h urine collections, based on creatinine excretion data. The mean daily 24-h urinary sodium excretion was 252.0 ± 92.2 mmol/day, equal to daily salt intake of 14.8 ± 5.4 g. Of the 657 participants with accurate 24-h urine collections, 464 (70%) provided fully completed dietary recalls. Among these 464 participants, there was a significant difference between the 24-h urinary sodium excretion-based salt intake estimation (14.5 ± 5.1 g/day) and the dietary recall-based salt intake estimation (12.0 ± 7.0 g/day) (*p* < 0.001). On the other hand, a positive correlation was obtained between the dietary recall-based daily salt intake and 24-h urinary sodium excretion-based daily salt intake (*r* = 0.277, *p* < 0.001). Bread was the main source of salt (34%) followed by salt added during cooking and preparing food before serving (30%), salt from various processed foods (21%), and salt added at the table during food consumption (11%). Conclusively, this study confirmed a very high salt intake of the adult population in four major cities in Turkey. The present findings support the emerging salt reduction strategy in Turkey by promoting lower salt content in baked bread, and less salt use in habitual food preparation and during food consumption in the home.

## 1. Introduction

The relationship between hypertension and dietary salt is well known, and has been documented in experimental, observational, and clinical studies [1,2,3,4]. It is also known that the amount of daily salt consumption is above the recommended limits in many countries [2,5]. Strategies focusing on decreasing dietary salt would decrease the prevalence of hypertension and the incidence of cardiovascular events [2,6]. In Finland and the United Kingdom, population-level reductions in salt intake have been associated with declines in rates of cardiovascular diseases [7,8]. A recent modelling study of preventable risk factors for coronary heart disease (CHD) in Turkey reported that dietary changes (i.e., less saturated fat, less salt and higher fruit/vegetable consumption) can make the greatest contribution in reducing the CHD burden, with salt reduction alone saving 17,000 lives/year and 28,000 lives/year by 2025 at salt intake levels of 10 g and 5 g per day, respectively [9]. In light of these data, determining the sources of dietary salt by examining eating habits of societies and raising awareness on this issue appear to be an important preventive measure for cardiovascular diseases. In developed countries, salt-restriction strategies are being established by revealing the main sources of dietary salt. Unfortunately, studies on the sources of dietary salt are limited in number in low- and middle-income countries with high burdens of hypertension and cardiovascular diseases. In Turkey, which is an upper-middle income country, cardiovascular risk is high and hypertension is prevalent. In a previous study, we showed that daily salt intake in Turkey was too high (18.01 g/day) [10]. The present study aimed to investigate dietary sources of such high salt consumption in Turkey, as knowing the sources of dietary salt will form the basis for preventive strategies aimed towards salt reduction.

## 2. Materials and Methods

This study was conducted on a sub-group selected from the population of the PatenT2 study [11] in 2012, which was representative of Turkey and conducted to determine the rates of prevalence, awareness, and control of hypertension in adults. The exclusion criteria for this study were as follows: being pregnant, using diuretics, fasting for the last 24 h before enrollment, and having cardiac failure, renal failure, chronic liver disease, or diabetes mellitus. The present study was designed to represent the adult population in the urban and rural areas of four major cities (Ankara, Istanbul, Izmir, and Konya). These four cities account for 1/3 of the country’s population, have a population distribution across urban and rural areas similar to that of country, and have a variety of dietary habits.

Sociodemographic characteristics and medical histories of all participants, as well as detailed records and histories of diet (content and amount) and salt consumption, were recorded via a questionnaire and a detailed face-to-face interview. A 24-h urine sample was collected from all participants within the period of February–March 2012 for the analysis of sodium, potassium, urea, and creatinine levels. An explanatory leaflet, along with the necessary equipment, was given to all participants, and they were instructed carefully about the method of urine collection. Beginning from the first urine sample of the day (in the morning), urine was collected over 24 h and was transferred into the urine container. Participants were instructed to keep urine samples in a cool and dark place. At the end of the collection period, healthcare workers measured the urine volume. The urine samples were then placed into cooler bags (4 °C) (Igloo Products Corporation, Katy, TX, USA) and within 24 h, they were sent to the central laboratory, where analyses were performed immediately. Participants who had 24-h urinary creatinine excretion within the predetermined limits of 10–30 mg/kg [12] were included in the analysis. Daily salt intake was estimated based on calculation of 24-h urinary sodium excretion on the assumption that all sodium ingested was in the form of sodium chloride. Salt intake was calculated using the equation of 1 g salt = 17.1 mmol of sodium in 24-h urine.

Each participant was instructed to record his/her food intake in detail (dietary recall) on the day (any day in a week) that he/she collected the 24-h urine sample. On the next day, the interviewers, who were trained for this study, visited the participants’ houses to collect the 24-h urine samples, checked the diet recalls, asked the participants to complete any omissions, and clarified any details as required. Thereafter, they questioned the participants about how many times and how much they eat a day; the amount of salt and tomato paste (which includes significant salt and is used in almost all home-made foods in Turkey) added during the meal preparation; and were also asked as to the household member preparing the meals. The accuracy of the forms tried to be achieved by a double check in the study. After the interviewers checked the forms, they marked some forms as inaccurate. Then, the researchers evaluated the dietary recall forms of the participants without knowing the interviewers’ interpretation. The researchers controlled whether the forms were adequately completed or not and they also marked some forms as inaccurate. Then, in the final check, all forms marked as inaccurate by the interviewers or researchers were excluded from the analysis.

In order to determine the amount of salt added during food consumption, a pre-estimation method was derived by the researchers. In this estimation, 12 different saltshakers were used, and the number and size of holes of each saltshaker were measured. These saltshakers were given to 12 individuals, and they were asked to add a sprinkle of salt using their saltshakers as they always do while eating. The amount of salt sprinkled on from each saltshaker was then measured in grams. The average amount of salt was calculated and this was accepted as the amount of salt consumed when a participant adds salt once during the meal. All participants reported how many times they added salt to each meal and the amount of salt consumed was calculated according to these estimates. All participants were also asked to record how much bread they ate (number and size of slices) and the amount of salt consumed from bread was calculated according to the estimation by Akpolat et al. [13]. The amount (grams per serving) of food eaten was coded from the Food and Nutrition Photograph Catalog [14]. The group of each food item recorded in the dietary recall of the participants was identified and, accordingly, the salt content of each food was estimated in grams per 100 g of food using the United States Department of Agriculture National Nutrient Database [15].

### 2.1. Sample Size

In the sample size calculation, inclusion of 249 participants was found to be adequate, with the assumption that the precision of the amount of salt calculated from the amount of sodium estimated from 24-h urine samples was 1 g and that the estimated standard deviation was ±8 with a two-sided 95% confidence interval at a significance level of 0.05. Since there was a difference between male and female participants in terms of eating habits, inclusion of 498 (2 × 249) participants was found to be adequate for two different clusters. Additionally, assuming a 30% loss related to the dietary recall assessment forms that were not fully completed, inclusion of 711 (249 × 2/0.7) participants was found to be adequate in the present study.

### 2.2. Statistical Analysis

All statistical analyses were performed using the Predictive Analytics Software (PASW 18.00; SPSS Inc., Chicago, IL, USA). In the comparison of normally distributed numeric and categorical variables, Student’s t-test and chi-square test were used, respectively. The correlation analysis for the relationship between the salt intake calculated by 24-h urinary sodium and the salt intake estimated by dietary recall was performed using Spearman’s rho test. The Bland-Altman method was also used to assess the agreement between salt intake calculated from the dietary recall and 24-h urine measurement [16]. All tests were evaluated at 5% type-I error level to infer statistical significance.

## 3. Results

Among 925 participants enrolled, data from 657 participants who had 24-h urinary creatinine excretion within the predetermined limits were analyzed. Of the participants, 53.1% were female and 46.9% were male. Demographic and clinical characteristics of the participants are presented in Table 1. The amount of 24-h urinary sodium excretion was 252.0 ± 92.2 mmol/day. The corresponding mean daily salt intake was 14.8 ± 5.4 g/day with a significant difference between males and females (15.7 ± 5.5 g/day and 14.0 ± 5.2 g/day, respectively; *p* < 0.001). Daily salt intake was higher in those living in the rural areas (*n* = 144 (22%)) compared with those living in urban areas (*n* = 513 (78%)) (16.0 ± 5.5 g/day vs. 14.5 ± 5.4 g/day, *p* = 0.001). There were no significant differences between urban and rural areas in terms of age (47.5 + 15.5 years vs. 48.1 + 14.7 years, *p* = 0.799), male/female ratio (231/282 vs. 77/67, *p* = 0.073), and body mass index (BMI) (28.8 + 5.9 kg/m^2^ vs. 29.2 + 5.3 kg/m^2^, *p* = 0.293).

There were 464 (70.6%) participants with fully completed dietary assessment forms. These participants were compared with the remaining 193 participants who did not record their diet correctly regarding age, gender, and BMI. There was no significant differences between the groups in terms of gender and BMI. The mean age of the participants correctly recording their dietary recall was higher (49 ± 15 vs. 44 ± 16, *p* = 0.001).

The mean estimated daily salt intake according to the dietary recall data was 12.0 ± 7.0 g/day (Table 2). While the dietary recall-based salt intake was similar in males and females (11.9 ± 6.6 and 12.2 ± 7.3, respectively; *p* = 0.950), salt intake was higher in those living in the rural areas, compared with those living in the urban areas (14.5 ± 8.1 and 11.3 ± 6.4, respectively; *p* = 0.001).

The absolute difference between the 24-h urinary sodium excretion-based salt intake estimation (14.5 ± 5.1 g/day) and the dietary recall-based salt intake estimation (12.0 ± 7.0 g/day) was about 2.5 g; the difference was statistically significant (*p* < 0.001). There was a positive correlation between the dietary recall-based salt intake estimation and the 24-h urinary sodium excretion-based salt intake estimation (*r* = 0.277, *p* < 0.001) in the participants with reliable dietary assessments, as well as both in males (*r* = 0.238, *p* < 0.001) and females (*r* = 0.310, *p* < 0.001) separately.

**Figure 1:**
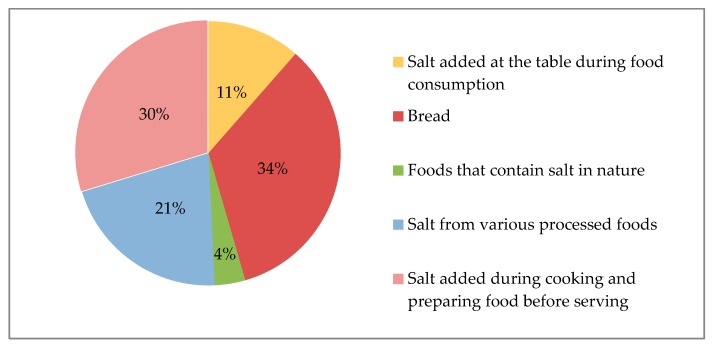
Sources of dietary salt.

Evaluation of the sources of salt in dietary recall revealed that the majority of salt consumed was from bread (34%) and salt added during food preparation (30%) followed by processed foods (21%) and salt added during food consumption (11%) (Table 3, Figure 1). Food items belonging to processed foods are also presented in Table 3. There were significant differences both between males and females (4.6 + 4.0 g/day vs. 3.7 + 4.0 g/day, *p* < 0.001) and between urban and rural areas (3.5 + 2.9 g/day vs. 6.3 + 6.1 g/day, *p* < 0.001) in terms of the amount of salt intake from bread.

**Figure 2:**
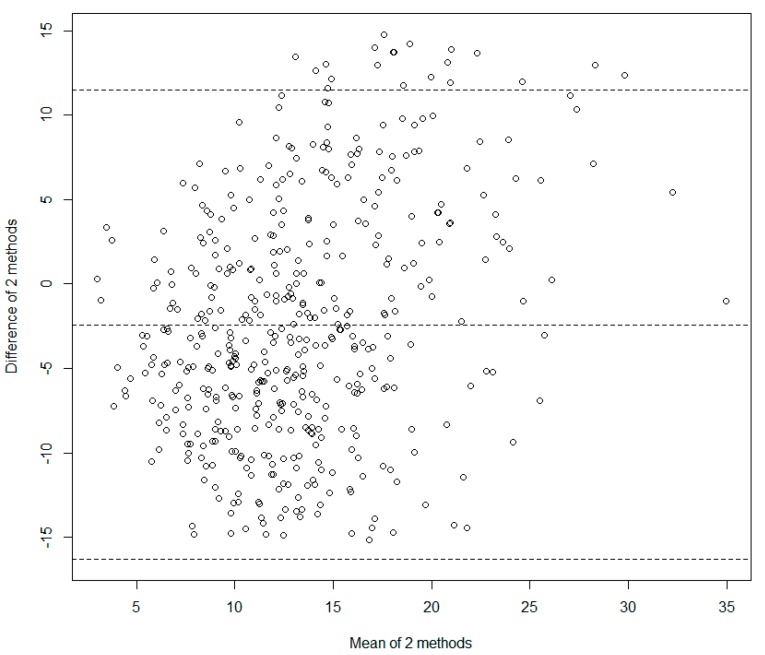
The Bland-Altman plot.

The limits of agreement for the difference were calculated as −16.579 g/day to 11.750 g/day using the Bland-Altman method (Figure 2). The mean difference and its 95% confidence intervals were −2.414 g/day (Confidence interval: −3.060 g/day to −1.768 g/day).

## 4. Discussion

It is already known that salt consumption is very high in Turkey. The present study determined once more that salt consumption was still high (14.8 salt g/day) even though it was lower than our finding of 18.0 g/day in 2007, reported in the previous study [10]. The present study also determined the sources of dietary salt in Turkey. It was found that bread was the main source (34%, 4.1 ± 4.1 g/day), followed by salt added during food preparation (30%, 3.6 ± 4.5 g/day), processed foods (21%, 2.5 ± 2.5 g/day), and salt added during food consumption (11%, 1.4 ± 2.5 g/day). The most striking point of this distribution is that salt intake was lower from processed foods, but higher from bread and traditional cooking style, compared to in developed countries [17,18].

Various methods can be used to determine the amount and sources of salt intake [19,20,21,22]. Collecting a 24-h urine sample is the gold standard method of determining the amount of salt intake for population surveys. The present study estimated the mean salt intake of the subgroup as 14.5 ± 5.1 g/day based on a single accurate 24-h urine collection. Dietary recall, which is used both to estimate the amount and to determine the sources of salt consumption, was also used in the present study and the salt consumption of the subgroup determined using dietary recall was 12.0 ± 7.0 g/day. However, there was a significant difference between daily salt intake estimated by the dietary recall and by the 24-h urinary sodium excretion (*p* < 0.001). This difference might be attributed to using a non-validated dietary recall questionnaire. However, the positive correlation obtained between the dietary recall-based salt intake estimation and the 24-h urinary sodium excretion-based salt intake estimation (*r* = 0.277, *p* < 0.001) in this study might suggest relative reliability of the data on the sources of salt determined based on the dietary recall. Some previous studies have also reported similar results [23]. Accordingly, the data of dietary recall assessments were used for the subgroup analysis of the food items in the present study.

Sources of dietary salt vary among countries. In the USA, nearly half (44%) of dietary sodium was obtained from 10 different categories of foods: bread and rolls, cold cuts/cured meats, pizza, poultry, soups, sandwiches, cheese, pasta mixed dishes, meat mixed dishes, and savory snacks [17]. A large-scale study from the USA evaluating 24-h diet in children, adolescents, and adults reported that approximately 1/3 of daily salt consumption was obtained from foods eaten outside the home; therefore, supermarkets, restaurants, and schools also had a role in reducing dietary salt [24]. In the present study, the main source of salt intake was bread (34%), followed by salt added during food preparation (30%) and salt intake from processed foods (21%). The main source of salt in South India was found to be salt added to the foods prepared with pulses, rice, vegetables, fruits, and milk and dairy products [25]. To our knowledge, data on sources of salt consumption from underdeveloped countries are limited.

In Turkey, bread (34%) was found to be the main source of dietary salt. In a review evaluating salt intake in Europe, bread contribution to total salt intake was reported as 25.9% in Ireland, 24.8% in Belgium, 24.2% in France, 19.1% in Spain, and 19% in the UK [26]. In another study from Portugal, the bread contribution to daily salt intake was reported to be 20–27% [27]. One potential reason for this difference may be the variance in the amount of salt in bread (502 mg/100 g bread in Spain, 708 mg/100 g bread in France, and 397 mg/100 g bread in the UK [26]) and the amount of bread consumed daily. According to a study by Akpolat et al. [13], the amount of salt (approximately 1800 mg) in 100 g bread and the daily average bread consumption (about 400 g/day/person) are very high in Turkey compared to other countries, for example, 186 g/day/person in Ireland, 129 g/day/person in Belgium, 137 g/day/person in France, 126 g/day/person in Spain, and 101 g/day/person in the UK [26]. Bread is the main source of dietary salt and it is suggested that a reduction in bread salt would have a significant impact on global health [28]. However, as salt is an important component of bread baking, salt reduction has some dimensions that could affect both producers and consumers, such as taste, volume, quality, and shelf-life [28]. Bread is a widely used nutritional element, particularly in middle-income countries. For this reason, the development of country-specific policies for reducing bread consumption and reducing salt in bread is necessary. Within this context, according to the regulations of the Republic of Turkey Ministry of Food, Agriculture and Livestock, the recommended salt content of bread in Turkey was changed in 2012 from 1.75 g/100 g bread to 1.5 g/100 g bread as part of a salt-reduction health initiative [29]. Even the food industry has not developed an action plan to reduce salt in bread; the government has planned to follow up this regulation, and has made attempts to inspect bread bakeries with respect to the amount of salt in bread. Although the average salt level of bread in the UK is lower than in Turkey, attempts to reduce the amount of salt in 100g of bread are ongoing; as a result, there was a reduction of approximately 20% between 2001 (1.23 ± 0.19 g/100 g) and 2011 (0.98 ± 0.13 g/100 g) in the UK [30]. Moreover, in the comprehensive overview of national initiatives for salt reduction by Webster et al. [31], attempts to reduce salt in bread were reported in many countries, such as a reduction by 26% in Spain between 2005 and 2009, a reduction by 18% in Ireland between 2003 and 2013, a reduction by 12% in France between 2008 and 2011, and a reduction by 6% in Belgium between 1990 and 2009. In the systematic review and meta-analysis by Jaenke et al. [32], the authors concluded that salt could be substantially reduced in bread, which was one of the highest contributors to population salt intake, without jeopardizing consumer acceptability.

The percentage of salt intake from processed foods (21%) was lower in the present study than for developed countries. Among the processed foods consumed in Turkey, foods for breakfast (62%), pickles (16%), and meat-poultry-fish and meat products (10%) were determined to be the main sources of salt. Frozen food products and prepared foods are not consumed widely in Turkey. However, consumption of frozen food products will increase with the increasing developmental level of the country, leading to an increased risk for future generations.

In various countries, ongoing efforts are being made to determine strategies and to develop nationally applicable models for popularizing salt restriction in order to reduce the amount of daily salt intake within the limits defined by the World Health Organization [33,34,35]. To achieve the intended goals, it would be necessary to know the current status of salt intake and the sources of salt depending on the eating habits of each population. For instance, data from the USA has revealed that the amount of salt intake from processed foods is very high (>80%); thus, what needs to be done in the USA is a reduction in salt in processed foods and in restaurants. Whereas in Turkey, reducing salt in bread and salt added in traditional cooking methods would be a more effective strategy for a nationwide salt reduction policy. Following the previous epidemiological study on salt consumption in Turkey [10], the Republic of Turkey Ministry of Health started a strategy program for salt reduction in 2011 [29,36]. In this action plan, firstly the salt contents of bread, pastrami, olives, cheese, and tomato paste were reduced. Secondly, saltshakers were removed from the tables of public cafeterias. Lastly, public service announcements were broadcast on television and regulations were developed for school canteens [36]. However, it has yet to be found whether government regulations are applied by the food industry and local bakeries. Moreover, there is a need to investigate the potential health impacts of this reduction at the population level. Currently, the Republic of Turkey Ministry of Health has announced the second term of the action plan covering the years 2017–2021. This plan has the aims for monitoring whether the salt content of bread and other food items have been reduced across the country. The health implications of salt reduction will also be evaluated during this action plan.

The strength of the present study was the inclusion of an adequate number of participants with accurate 24-h urinary samples and fully completed dietary recall assessment forms. However, collecting 24-h urinary sample and dietary recall data from the participants only once was the main limitation of the present study. Another limitation was not using a validated dietary recall questionnaire. We observed that the difference between the daily salt intake estimated by the dietary recall and estimated by the 24-h urinary sodium excretion was large, which was also a limitation of the present study. Thus, one should consider that the distribution of dietary salt sources presented is valid with the assumption of a non-selective discordance for the different sources of salt between the two measurements. Bearing this in mind, we still believe that this distribution at a minimum correctly describes the relative dominance of bread and added salt during food preparation as the commonly preferred sources. In further field studies, it would be beneficial to collect 24-h urinary samples more than once at different times, as well as to use a validated dietary recall questionnaire at the day 24-h urinary samples will be collected.

## 5. Conclusions

In conclusion, mean adult daily salt consumption is far above recommendations in Turkish cities, as it is in many regions of the world. Moreover, relatively high bread consumption and the corresponding high salt intake in Turkey were remarkable outcomes of the present study. Each country needs to plan and take strategical measures appropriate for their own eating habits. For this purpose, health authorities, legislators, and food producers should collaborate and develop models appropriate for their countries. The findings of this study of the main sources of dietary salt will help further developments of salt reduction strategies in Turkey.

## Figures and Tables

**Table 1:** Demographic and clinical characteristics of the 657 participants with accurate 24 h urine samples.

Variable	Male (*n* = 308)	Female (*n* = 349)	Total (*n* = 657)	*p*
**Age, years, mean ± SD**	48.7 ± 16.1	46.7 ± 14.6	47.6 ± 15.3	0.073
**Age groups, years, *n* (%)**				
18–35	75 (24.4)	90 (25.8)	165 (25.1)	0.308
36–64	187 (60.7)	221 (63.3)	408 (62.1)	
≥65	46 (14.9)	38 (10.9)	84 (12.8)	
**BMI, kg/m^2^, mean ± SD**	27.9 ± 4.6	29.8 ± 6.5	28.9 ± 5.7	0.001
BMI groups, *n* (%)				
Normal weight (<24.9 kg/m^2^)	84 (27.2)	102 (29.2)	186 (28.3)	<0.001
Overweight (25–29.9 kg/m^2^)	125 (40.6)	92 (26.4)	217 (33.0)	
Obese (≥30 kg/m^2^)	99 (32.1)	155 (44.4)	254 (38.7)	
**Hypertension, *n* (%)**				
Absent	184 (59.7)	218 (62.5)	402 (61.2)	0.475
Present	124 (40.3)	131 (37.5)	255 (38.8)	
**BP, mmHg, mean ± SD**				
Systolic BP	131.0 ± 19.5	123.2 ± 19.2	126.9 ± 19.7	<0.001
Diastolic BP	75.4 ± 11.5	72.1 ± 10.8	73.7 ± 11.2	<0.001
**Laboratory parameters, mean ± SD**				
24-h urinary sodium, mmol/day	267.4 ± 94.2	238.4 ± 88.3	252.0 ± 92.2	<0.001
Urinary creatinine, mg/day	1746.9 ± 486.2	1373.5 ± 407.3	1548.5 ± 483.1	<0.001

SD, standard deviation; BMI, body mass index; BP, blood pressure.

**Table 2:** Salt intake in a subset of 464 participants with fully completed dietary assessment forms and a valid 24-h urine collection.

	Male (*n* = 210)	Female (*n* = 254)	Total (*n* = 464)
24-h urinary sodium, mmol/day	256.4 ± 85.6	237.0 ± 87.4	245.8 ± 87.0
Salt intake estimated from 24-h urinary sodium, g/day	15.1 ± 5.0	13.9 ± 5.1	14.5 ± 5.1
Salt intake estimated from dietary recall, g/day	11.9 ± 6.6	12.2 ± 7.3	12.0 ± 7.0

**Table 3:** Estimated dietary salt intake (g/day) and distribution of major food sources of salt.

Food Items	Estimated Salt Intake (g/Day, Mean ± SD)
Bread	4.1 ± 4.1
Salt added during cooking and preparing food before serving	3.6 ± 4.5
Salt from various processed foods	2.5 ± 2.5
	**Distribution of processed foods (%)**
Breakfast foods (cheese, olive, butter, eggs, etc.)	62%
Pickle	16%
Meat-poultry-fish and meat products	10%
Dried nuts and fruits	4%
Biscuits and crackers	3%
Others *	6%
	**Estimated Salt Intake (g/day, Mean ± SD)**
Salt added at the table during food consumption	1.4 ± 2.5
Foods that contain salt in nature (e.g., some vegetables, etc.)	0.5 ± 0.8

SD, standard deviation; * Frozen food products and ready-to-eat food products.
